# Supplementary material for: Identification of Morphologic Criteria Associated with Biochemical Recurrence in Intraductal Carcinoma of the Prostate
Source: Cancers (Basel). 2021 Dec 13;13(24):6243. doi: 10.3390/cancers13246243 (PMC8699439; doi:10.3390/cancers13246243)
Supplement: Supplementary file 1 [file cancers-13-06243-s001.zip › cancers-1463893-supplementary.pdf]

Supplementary Materials

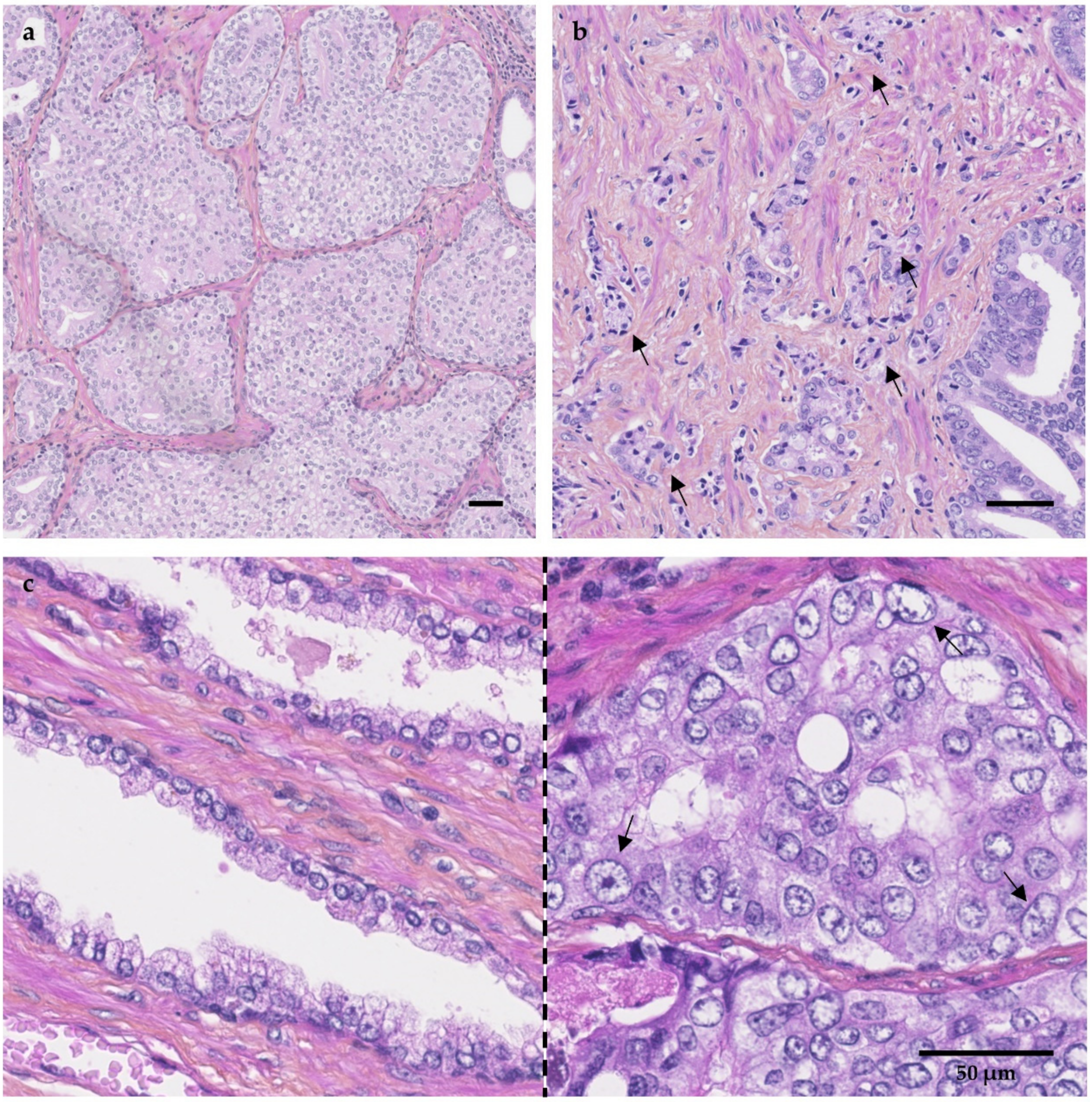

Figure S1. Other morphologic criteria evaluated. (**a**) Solid pattern. (**b**) Pyknotic nuclei (arrows) in the adjacent invasive cancer. (**c**) Nuclei size, nuclei in benign glands (left), and >180 μm^2 nuclei in IDC-P (right, arrows) on the same slide. HPS staining. Scale bars: 50 μm.

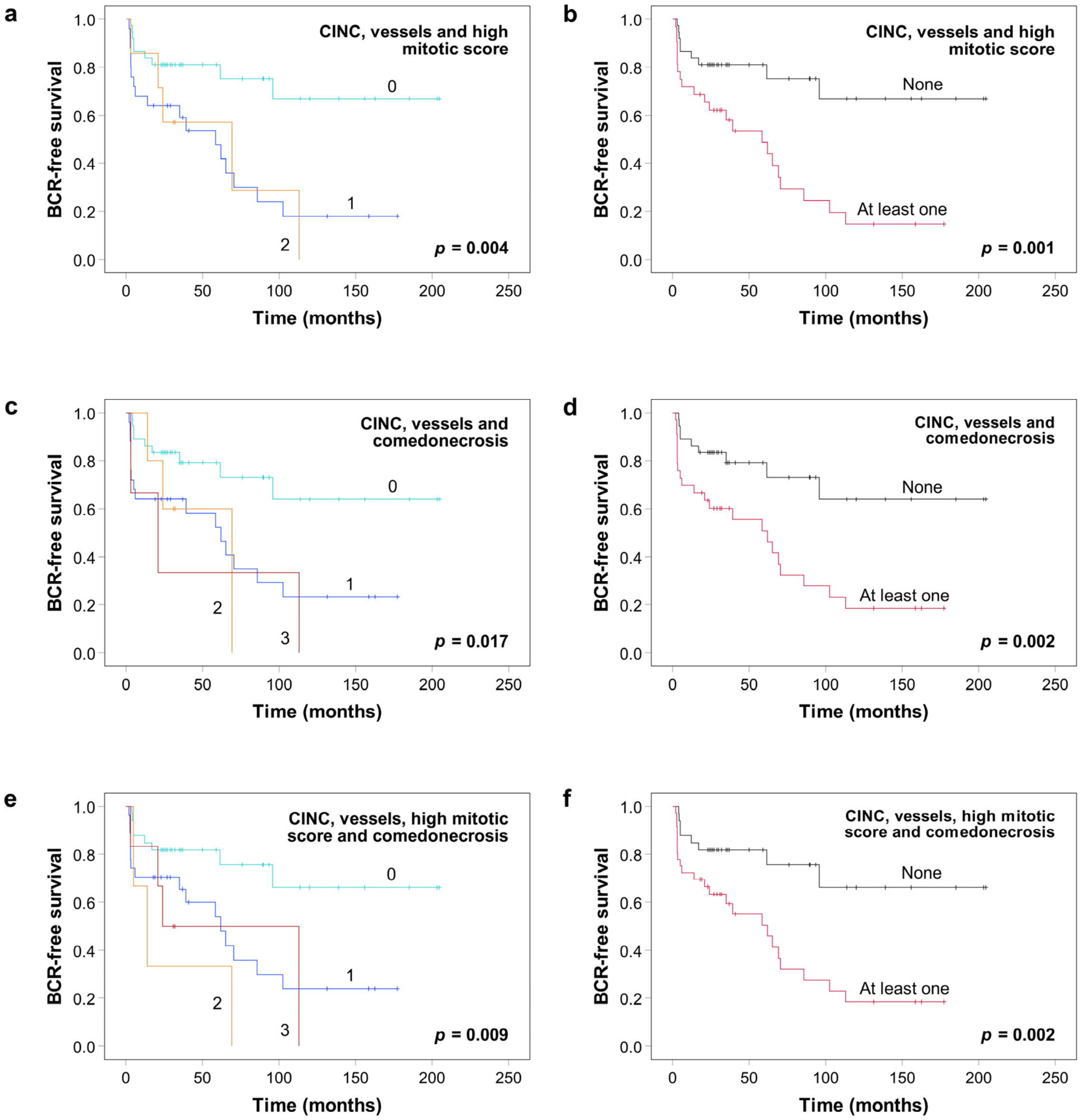

Figure S2. Kaplan–Meier curves of BCR-free survival according to the number (**a**,**c**,**e**) or the presence or absence (**b**,**d**,**f**) of three to four proposed criteria for IDC-P in the validation cohort. *p*-values were calculated using the log-rank test. CINC: Cells with irregular nuclear contours.

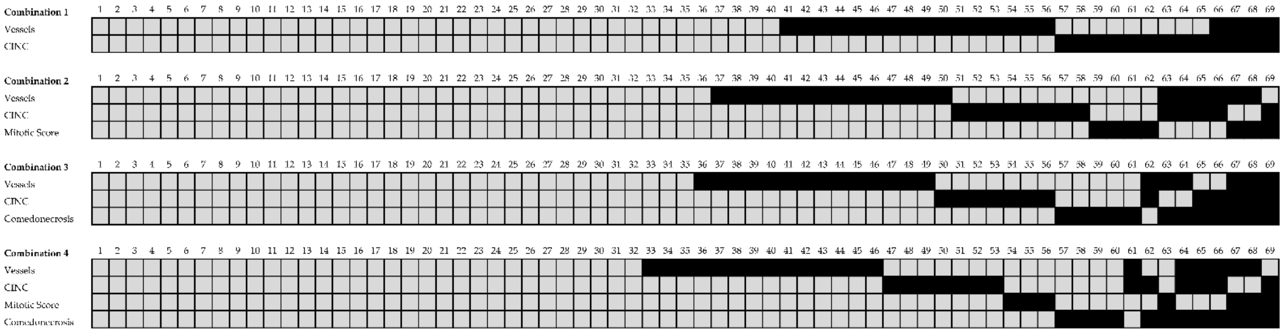

Figure S3. Distribution of criteria according to the four combinations of criteria in the validation cohort. Each column represents a patient and each line a criterion. Adverse criteria are represented by black squares. CINC: cells with irregular nuclear contours.

Table S1. Cox regression analysis for the prediction of BCR in the validation cohort according to the presence of three to four proposed criteria for IDC-P.

Included Criteria	Variables	Validation Cohort (n = 69)					
		Univariate Analysis			Multivariate Analysis		
		HR	95% CI	*p*-Value	HR	95% CI	*p*-Value
CINC, vessels and mitotic score	Grade group *						
	1–2	ref			*ref*		
	3	2.72	1.07–6.92	**0.036**	2.13	0.82–5.51	0.121
	4–5	5.05	1.96–13.02	**0.001**	3.77	1.42–10.00	**0.008**
	Number of adverse criteria						
	0	ref					
	1	3.44	1.53–7.73	**0.003**			
	2	3.50	1.17–10.49	**0.025**			
	0 vs. ≥ 1	3.45	1.59–7.51	**0.002**	2.64	1.18–5.89	**0.018**
CINC, vessels and comedonecrosis	Grade group *						
	1–2	ref			*ref*		
	3	2.72	1.07–6.92	**0.036**	2.35	0.92–6.02	0.074
	4–5	5.05	1.96–13.02	**0.001**	4.27	1.64–11.11	**0.003**
	Number of adverse criteria						
	0	ref					
	1	3.04	1.34–6.88	**0.008**			
	2	3.21	0.86–12.01	0.084			
	3	4.43	1.19–16.42	**0.026**			
	0 vs. ≥ 1	3.20	1.47–6.95	**0.003**	2.70	1.23–5.91	**0.013**
CINC, vessels, mitotic score and comedonecrosis	Grade group *						
	1–2	ref			*ref*		
	3	2.72	1.07–6.92	**0.036**	2.36	0.92–6.02	0.074
	4–5	5.05	1.96–13.02	**0.001**	4.18	1.60–10.89	**0.003**
	Number of adverse criteria						
	0	ref					
	1	2.99	1.28–6.98	**0.012**			
	2	5.97	1.56–22.86	**0.009**			
	3	3.64	1.09–12.16	**0.036**			
	0 vs. ≥ 1	3.30	1.47–7.38	**0.004**	2.74	1.21–6.19	**0.015**

HR: hazard ratio; CI: confidence interval; CINC: cells with irregular nuclear contours. * Grade groups 1–2 and 4–5 were combined because of the small number of patients in grade groups 1 (n = 5) and 4 (n = 5). Bold entities indicate statistically significant *p*-values.

Table S2. Association of each proposed criteria with grade group, pT stage, and surgical margin status in the test and validation cohorts.

Factor	Adverse Criteria	Test Cohort (n = 39)			Validation Cohort (n = 69)		
		n (%)	Mean Rank	*p*-Value	*n* (%)	Mean Rank	*p*-Value
**Grade Group**	**CINC**			**0.035** [a]			0.831 [a]
	Absence	31 (79)	18.16		56 (81)	35.23	
	Presence	8 (21)	27.13		13 (19)	34.00	
	**Vessels**			**0.027** [a]			0.097 [a]
	Absence	32 (82)	18.22		49 (71)	32.61	
	Presence	7 (18)	28.14		20 (29)	40.85	

	**Mitotic score**			**0.044** [a]			0.097 [a]
	Low	29 (74)	17.97		62 (90)	33.74	
	High	10 (26)	25.90		7 (10)	46.14	
	**Comedonecrosis**			0.116 [a]			0.912 [a]
	Absence	28 (72)	18.30		57 (83)	34.89	
	Presence	11 (28)	24.32		12 (17)	35.54	
**pT stage**	**CINC**			0.517 [a]			0.628 [a]
	Absence	31 (79)	19.47		56 (81)	34.47	
	Presence	8 (21)	22.06		13 (19)	37.27	
	**Vessels**						0.132 [a]
	Absence	32 (82)	19.34	0.385 [a]	49 (71)	32.83	
	Presence	7 (18)	23.00		20 (29)	40.33	
	**Mitotic score**			**0.029** [a]			0.273 [a]
	Low	29 (74)	17.93		62 (90)	34.17	
	High	10 (26)	26.00		7 (10)	42.36	
	**Comedonecrosis**			0.050 [a]			0.183 [a]
	Absence	28 (72)	18.02		57 (83)	33.62	
	Presence	11 (28)	25.05		12 (17)	41.54	
**Margin status**	**CINC**			0.394 [b]			0.725 [b]
	Absence	31 (79)	N/A		54 (81)	N/A	
	Presence	8 (21)			13 (19)		
	**Vessels**			0.403 [b]			0.570 [c]
	Absence	32 (82)	N/A		47 (70)	N/A	
	Presence	7 (18)			20 (30)		
	**Mitotic score**			0.693 [b]			1.000 [b]
	Low	29 (74)	N/A		60 (90)	N/A	
	High	10 (26)			7 (10)		
	**Comedonecrosis**			0.262 [b]			0.483 [b]
	Absence	28 (72)	N/A		55 (82)	N/A	
	Presence	11 (28)			12 (18)		

CINC: cells with irregular nuclear contours. N/A: not applicable. Bold entities indicate statistically significant *p*-values. [a] Mann–Whitney *U* test; [b] Fisher's exact test; [c] Pearson chi-square.
